# Bringing anatomy to life: the role of clinical ultrasound in undergraduate medical education – a systematic review

**DOI:** 10.1186/s13089-025-00443-3

**Published:** 2025-08-07

**Authors:** Mathilde Brunel, Valentin Sebastian Schäfer, Florian Recker

**Affiliations:** 1 Clinic of Internal Medicine III Department of Oncology, Hematology, Rheumatology and Clinical Immunology, University Hospital Bonn, Bonn, Germany; 2 Department of Obstetrics and Prenatal Medicine, University Hospital Bonn, Bonn, Germany

**Keywords:** Clinical ultrasound, Anatomy learning, Medical student, Curriculum, Educational outcome

## Abstract

**Background:**

The implementation of ultrasound as point-of-care technique within the medical education has been broadly studied to understand its applicability in clinical settings. More research has been conducted to specifically understand the use of clinical ultrasound in the medical curriculum to learn anatomy. The results of publications on anatomical skills and knowledge outcomes of students related to ultrasound are contradictory. The objectives of this study were to identify and describe common study types and educational programs conducted in anatomy teaching with clinical ultrasound and to describe the impact on learning outcomes.

**Methods:**

A literature search from the databases Scopus, PubMed and Google scholar was conducted with keywords related to anatomy learning, ultrasound and medical education. Data from publications were extracted following quantitative and qualitative methods previously described in the literature and adapted to measure the educational outcome.

**Results:**

In total, 1615 records were detected within all three databases after removing duplicates, 194 were found relevant to the topic and included in the review. Of 194, 128 articles were original studies categorized by their study types and outcomes. A large proportion of the studies were conducted at a single institute, and students were mainly evaluated with post-tests only or with pre- and post-tests. Vascular and cardiac anatomical landmarks were the most frequently instructed areas with ultrasound while ocular and prostate/testicular landmarks were the least. Students agreeing to participate in the ultrasound training were highly motivated and described the sessions as valuable for future clinical practice. The evaluation of anatomical knowledge and skills of students following ultrasound training varied widely, and no clear consensus emerged. Long-term assessments of ultrasound-related anatomy competencies were notably underrepresented in the reviewed studies.

**Conclusions:**

The use of clinical ultrasound in undergraduate anatomy education is implemented through diverse teaching formats and is widely appreciated by medical students. However, standardized methods to assess anatomical understanding of medical students through ultrasound are lacking, impeding the comparison of educational outcomes across studies. Further research is needed to evaluate the long-term retention of skills and knowledge to better determine the effectiveness of ultrasound-based teaching.

**Supplementary Information:**

The online version contains supplementary material available at 10.1186/s13089-025-00443-3.

## Introduction

Anatomy is a fundamental component of medical education and has evolved from traditional cadaver-based dissection and teacher-centered lectures to more student-centered approaches. Recent anatomy instructions incorporate concepts of living anatomy such as body painting, peer physical examination, and the use of live models [1, 2]. To respond to the increasing clinical complexity of medical cases, the medical curriculum was reformed three decades ago from discipline, individual care based to community, comprehensive, personalized and integrated care within basic science and clinical practice [3, 4]. With increasing knowledge in medicine and fast technological advancements, medical imaging is put forward in anatomical education as relevant techniques to improve clinical practice [5]. Computer tomography, magnetic resonance imaging and ultrasound diagnostic tools are valuable instructional methods in medical education to reveal anatomical characteristics of the human body in vivo in two or three-dimensions and their natural variations [1]. Ultrasound is a non-invasive imaging technique allowing visualization of anatomical and pathological structures, therefore is an appropriate method to teach clinical care to medical students [6]. Ultrasound has been incorporated as a teaching tool in medical education for over two decades [6, 7]. The European Federation of Societies for Ultrasound in Medicine and Biology (EFSUMB) recommends the systematic implementation of ultrasound within the medical curriculum for teaching purposes in pre-clinical and clinical settings [8]. EFSUMB endorses the use of ultrasound to enhance general understanding of medical students in anatomy, physiology and pathology [8]. Pedagogical resources, strategies, objectives and clinical practice guidelines to implement ultrasound in medical education are available from EFSUMB [9]. However, information on specific standardized procedures related to the teaching and assessment methods is limited [8, 9].

Few systematic reviews have focused on the topic of ultrasound use in medical education to learn anatomy with different objectives, search methods and databases [10–12]. The review [10] completed a broad search for available publications, measured their quality and outcomes. One study focused on teaching methods, outcomes, skills and equipment at one geographical area (United Kingdom) and with the specific publication timeline of 2003–2022 [11]. Another review performed a search on basic concepts of anatomy and physiology learning with ultrasound, evaluating outcomes and teaching efficacy [12]. This study is a single systematic review to bring further knowledge on the application of ultrasonography in undergraduate anatomy courses from a large number of publications while combining established quantitative and qualitative methods. The study provides an overview of the clinical ultrasound implementation in anatomy learning at the undergraduate stage of medical education to understand common educational designs and their outcomes on anatomy competencies linked to future medical practice. Evidence was collected from articles published in the last ten years using items from the medical education research study quality instrument (MERSQI) [13] and the Kirkpatrick´s model [14] to identify trends and provide information for improvement.

## Methods

### Research questions and objectives

The objectives of the systematic literature review were to identify and describe the educational outcomes of anatomy learning of medical students associated with the use of clinical ultrasound.

The following questions were formulated to develop the search for articles:

- Can the inclusion of clinical ultrasound to the anatomy curriculum in medical education modify the learning outcome of anatomical structures?
- What are the opinions of medical students in using an ultrasound tool in anatomy courses?
- What are the challenges and opportunities when implementing clinical ultrasound in anatomy curriculum for medical students?

### Search strategy

This study was conducted following the Preferred Reporting Items for Systematic Reviews and Meta-Analyses (PRISMA) statement to explain the methodic revision of published articles and limit risks of bias [15]. The PRISMA 2020 is a 27-item checklist guiding authors on how to report article review methods and findings to ensure transparent, complete and accurate systematic reviews [15]. The focus of the study was defined following the “PICO” tool as Population, Intervention, Comparison, and Outcomes (Table 1). Other tools exist such as “SPIDER” (Sample, Phenomenon of Interest, Design, Evaluation, Research types) and “PICOS” (Population, Intervention, Comparison, and Outcomes, Study design) [16]. The search tools SPIDER and PICOS were considered overly limiting by authors in finding relevant articles and excluded. The use of PICO tool is recommended for a fully comprehensive literature review search [16].

**Table 1:** PICO search tool to define the research question

*P* (patient, population, problem)	Medical students learning anatomy
I (intervention, prognostic factor, exposure)	Ultrasound use as an anatomy learning tool
C (comparison)	Between diagnostic learning tools and anatomical areas
O (outcomes)	Evaluation methods; learning outcomes; student perceptions, attitudes and feedback; assessment of long-term retention.

A first search for articles relevant to the topic of ultrasound utilization in anatomy education was performed with the following keywords in Scopus: “curriculum” AND “anatomy” AND “ultrasounds”. A second more developed search with the keywords “anatomy” AND “ultrasounds” AND “medical” AND “students” was implemented in the databases Scopus, PubMed and Google scholar. All databases were last consulted on 07.02.2025. The authors focused on English language articles from 2014 to February 2025. The complete search results for each database can be provided upon request from the corresponding author.

### Inclusion and exclusion criteria

Prior to the selection of articles, we considered specific inclusion and exclusion criteria to address the research questions. As an inclusion criterium, studies targeting undergraduate medical students at all stages of education were accepted. Studies describing medical students and residents were accepted under the condition of clear data and findings separation. There was no exclusion based on the country of publication. Posters, conference papers, abstracts, editorials and letters to editor, non-peer-reviewed studies were excluded. Non-open access articles falling outside the University Hospital Bonn journal subscriptions were requested for access on ResearchGate and excluded in case of non-response.

### Screening

**Fig. 1:**
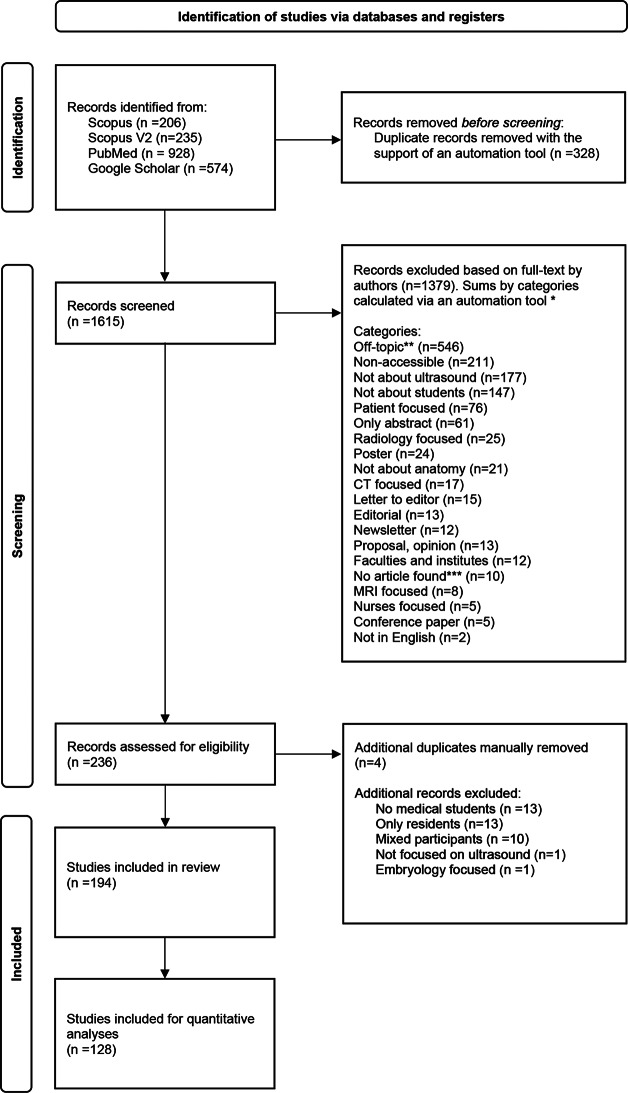
Flow diagram of the article selection process according to PRISMA *The total number of articles excluded and the number per category are not equal as some articles were assigned to multiple categories (for example, articles focused on patients and not about medical students). **Off-topic: articles not about ultrasound, anatomy, medical students and education. ***No article found: title of article shown in the database and text not available online. Abbreviations: CT, Computer tomography; MRI, Magnetic resonance imaging

Duplicates articles within and between databases were automatically detected with the support of the automation tool Rayyan and manually screened before removal from the systematic literature review by MB. In the next step, each article was retrieved as full-text for screening. All articles were manually screened and sorted by M.B. and F.R. Articles out of the review scope were categorized following a coding system: “off-topic”, “non-accessible”, “no ultrasound”, “not about medical students or residents”, “patient focused”, “only abstract”, “poster”, “computer tomography focused”, “letter to editor”, “radiology focused”, “editorial”, “newsletter”, “faculty and institute level”, “no article found”, “magnetic resonance imaging focused”, “nurses focused”, “no anatomy”, “conference paper”, “not in English”, “proposal or opinion paper”, “only medical residents”, “mixed participants”, embryology focused”. Excluded articles were added to multiple categories depending on their topics. The coding system was developed to ensure systematic sorting and was not implemented for statistical purposes. The complete search process is summarized in Fig. 1.

### Data extraction and analysis

All studies included after the screening process were investigated for their characteristics (type of study, demographics, outcome). Student attributes (year of medical education, group size) and course design (lecture/practical session hours, anatomical area studied) were quantified in the original studies.

The authors assessed the content of original articles included in the review using MERSQI [13]. Non-original studies were not analyzed with MERSQI as the instrument is specifically designed to assess the quality of methods implemented in educational research for original, quantitative studies [13]. Qualitative studies vary to a great extent in their design, sampling, evaluations and analyses compared to quantitative studies, requiring a different instrument [13]. The instrument contains 10 items grouped by categories such as study design, sampling, type of data, validity of evaluations instruments´ score, data analysis and outcome [13]. “Response rate” (item 3) was defined by authors as rate of medical students performing ultrasound training and tests after agreeing to participate. Type of data (item 4) included “Assessment by study subject” and “Objective measurement” to estimate the proportion of objective observation-based evaluations by teachers, trainers and peer-tutors in the original articles. Items 5, 6 and 7 within the validity category were evaluated based on definitions from [13] and [17] to measure internal consistency reliability of each study, relevance of the evaluation content to assess medical students and relationships between variables. “Patient and health outcome” term (item 10) was designated as the inclusion of real patients or standardized patients in the training program as well as any content related to direct patient health. Item 10 of the MERSQI is based on the Kirkpatrick´s model [14] as explained further below.

**Fig. 2:**
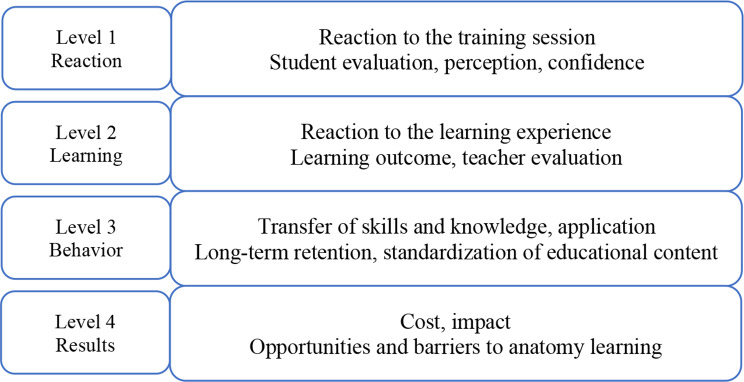
The Kirkpatrick´s model to evaluate the effectiveness of anatomy learning

To evaluate the effectiveness of the training program and qualitative results of the studies, the Kirkpatrick´s model was selected [14, 18]. The model is based on various factors (needs, objectives, appropriate facilities/instructors) and evaluation systems to plan and implement an effective training program. The four different levels, reaction, learning, behavior and results, are part of the model and were adapted to this systematic review and its objectives, as seen in Fig. 2.

The first level of the Kirkpatrick´s model measures the extent of the reaction from students, learners to the teaching program and materials [14]. Likert-scale is a method of choice to measure satisfaction and perception of students. In our study, we evaluated questionnaires and surveys described in the selected publications.

The second level of the model explains the degree of knowledge, skills and attitude change acquired during the training [14]. Types of tests (pre/post), teacher evaluations and Objective Structured Clinical Examination (OSCE) were considered in our study.

The third level is characterized as behavior or the magnitude of change in behavior related to participation in training programs [14]. We examined the long-term retention of knowledge and skills of medical students and reviewed the potential standardization of training programs.

The last and fourth level of the Kirkpatrick´s model represents the overall impact of a training program on all participants and includes practical questions such as time saved and cost estimations [14]. In our study, cost, future changes to a program and recommendations from authors for further applications were taken into account.

### Statistics

All statistical analyses presented in this study were performed in Microsoft Excel file (Version 2502, Microsoft 365, Microsoft Corporation, Redmond Washington, USA) and were presented as descriptive. Due to the heterogeneity of the articles, no formal meta-analysis could be implemented with size-effects, and some results rose above 100% due to multiple choices within each category.

## Results

### Search results

The literature search resulted in a total number of 1,943 records, of which 328 were removed after identification of duplicates (Fig. 1). From 1615 potential full-text-records screened, 1379 were categorized based on specific exclusion criteria (Fig. 1) and excluded. Four additional duplicates were detected and removed after further screening. A group of 38 records were excluded for not complying with all inclusion criteria. A total of 194 studies were considered relevant to the topic and were included in the literature review (Fig. 1).

### Study characteristics

Among the 194 included studies, more than half were original articles as shown in Table 2 Different types of reports (technical, medical, educational and brief) were published on the subject, indicating a variety of methods to evaluate and record teaching outcomes.

Half of the studies included in the review were conducted in the USA (50.5%), and to a lower degree in Germany (7.2%), UK (5.2%) and Canada (4.1%). Based on the affiliation information, the majority of countries across the world published their results as an individual university/institute/center, and few countries conducted collaborative work (6.2%).

**Table 2:** Characteristics of all studies selected in the systematic literature review (*N* = 194)

Characteristic	*N* (%)
Type of studies	
Original Paper	128 (66.0)
Review	23 (11.9)
Research report	13 (6.7)
Book, book chapter	4 (2.1)
Medical/educational report	4 (2.1)
Technical report	4 (2.1)
Study categories below 2%	
Method paper, case report, brief report, original report, descriptive article, overview, commentary, short communication, white paper	18 (9.3)
**Country**	**N (%)**
United States	98 (50.5)
Germany	14 (7.2)
United Kingdom	10 (5.2)
Canada	8 (4.1)
Category equal or less than 2% for each country	
Australia, Austria, Belgium, Brazil, Chile, China, Cyprus, Czech Republic, Egypt, France, Greece, India, Italy, Ireland, Israel, Japan, Korea, Mexico, New Zealand, Netherlands, Nicaragua, Nigeria, Romania, Rwanda, Saudi Arabia, South Africa, Spain, Switzerland, Taiwan, Thailand, United Arab Emirates	52 (26.8)
Collaborations	
Brazil, Canada, China, Egypt, Germany, Greece, Grenada, Ireland, Mexico, New Zealand, Poland, Rwanda, Switzerland, UK, USA	12 (6.2)

Percentages may not total 100 due to rounding

### Research study designs

The classification of original studies based on criteria from MERSQI showed that medical students were in majority enrolled from a single institution at 90.6% (item 2, Table 3). One reason could be the need to apply for ethical approval at the Institutional Review Board for each institution as seen in [19] and [20]. The recruitment of medical students from different programs has shown benefits with the possibility to compare an innovative curriculum from one program with a traditional curriculum from the other program [21]. The selected original studies were mainly conducted as a single group of students evaluated with post-tests (35.9%) or as a single group of students assessed with pre- and post-tests (28.9%) (item 1, Table 3), indicating a lack of a separate control group. The motivation of students after agreeing to participate was high with 64.8% response rate equal to or above 75% (item 3, Table 3).

The validity of evaluation instruments´ score was very low for all items (items 5, 6 and 7, Table 3), indicating low report of internal consistency between the assessment and the construct intended to evaluate, low report of content validity to assess the construct and little comparison between variables or a single variable to explain learning outcomes of medical students.

The statistical analysis of data (items 8 and 9, Table 3) was predominantly appropriate for the study design or the type of data and beyond descriptive analysis as many studies compared the student performance, skills and knowledge in identifying anatomical regions with ultrasound before and after teaching. Descriptive statistical analyses were often presented as percentages from surveys and questionnaires to illustrate satisfaction, perception and confidence levels of medical students.

Close to a third of the original articles measured satisfaction, perceptions, attitudes of medical students (item 10, Table 3) while behavior was the least studied (6.25%). In addition to the self-assessment of students, many of the publications evaluated the knowledge and skills assessments of medical students by teachers and trainers (43.8%).

**Table 3:** Study designs of original articles according to criteria from the MERSQI score [13]

Domain	MERSQI Item	*n*. (%)	Item score	Max score	Domain score Mean (SD)
Study design	*1. Study design*			3	1.66 (0.71)
	Single group cross-sectional or single group post-test only	46 (35.9)	1		
	Single group pre- and post-test	37 (28.9)	1.5		
	Non-randomized, 2 group	20 (15.6)	2		
	Randomized controlled trial	22 (17.2)	3		
	Non-determined	3 (2.34)	-		
Sampling	*2. Number of institutions studied*			3	1.72 (0.57)
	Single institution	116 (90.6)	0.5		
	Two institutions	8 (6.25)	1		
	More than 2 institutions	4 (3.13)	1.5		
	3. *Response rate*				
	< 50% or not reported	15 (11.7)	0.5		
	50–74%	16 (12.5)	1		
	≥ 75%	83 (64.8)	1.5		
	Not applicable	14 (10.9)	-		
Type of data	4. *Type of data*			3	2.03 (1.00)
	Assessment by study participant	59 (46.1)	1		
	Objective measurement	63 (49.2)	3		
	Non-determined	6 (4.69)	-		
Validity of evaluation instrument	*5. Internal Structure*			3	0.67 (0.76)
	Not reported	105 (82.0)	0		
	Reported	17 (13.3)	1		
	Not applicable	6 (4.69)	-		
	*6. Content*				
	Not reported	87 (68.0)	0		
	Reported	35 (27.3)	1		
	Not applicable	6 (4.69)	-		
	*7. Relationship to other variables*				
	Not reported	92 (71.9)	0		
	Reported	30 (23.4)	1		
	Not applicable	6 (4.69)	-		
Data analysis	8. *Appropriateness of analysis*			3	2.52 (0.71)
	Data analysis inappropriate for study design or type of data	30 (23.4)	0		
	Data analysis appropriate for study design or type of data	94 (73.4)	1		
	Not applicable	4 (3.13)	-		
	9. *Complexity of analysis*				
	Descriptive analysis only	29 (22.7)	1		
	Beyond descriptive analysis	95 (74.2)	2		
	Not applicable	4 (3.13)	-		
Outcome	10. *Outcome*			3	1.70 (0.72)
	Satisfaction, attitudes, perceptions, opinions, general facts	36 (28.1)	1		
	Knowledge, skills	56 (43.8)	1.5		
	Behaviors	8 (6.25)	2		
	Patient/health outcome	26 (20.3)	3		
	Non-Determined	2 (1.56)	-		
Total		128 (100)	-	18	10.3 (2.48)

Abbreviation: MERSQI, medical education research study quality instrument

### Participants and training characteristics

The population of medical students presented in the original articles was mainly first- and second-year students (Table 4). The percentage of students evaluated in anatomy skills with ultrasound decreased with increasing education (Table 4). These results should be considered as trends since 14.1% of the studies have not clearly specified the level of education of medical students. Nevertheless, these results support the idea that most anatomical courses are basic requirements and planned at the beginning of the medical curriculum. The duration of anatomy training with clinical ultrasound is mainly in hours with almost half of the studies including a study time between 60 min and 23 h while close to a third of the studies have not specified (Table 4).

**Table 4:** Learning parameters of the original articles selected (*n* = 128)

Parameter	*n* (%) *
Medical Year	
Year 1 Year 2 Year 3 Year 4 Year 5 Year 6 Nd	62 (48.4) 37 (28.9) 24 (18.8) 20 (15.6) 12 (9.4) 6 (4.7) 18 (14.1)
Training duration	**n (%)**
Up to 59 min From 60 min to 23 h From 1 to 6 days From 1 to 3 weeks From 1 to 11 months Years Nd	13 (10.2) 57 (44.5) 8 (6.3) 4 (3.1) 6 (4.7) 3 (2.3) 37 (28.9)

Abbreviation: Nd, non-determined

Percentages may not total 100 due to rounding

*The total n and total percentage (%) are above *n* = 128 and 100% as most studies included students from different medical years of education

The average group size of students during an ultrasound training was 139 individuals (Table 5), with a minimum group of 6 students [22] and a maximum sample size of 1260 learners in a study conducted for 6 years [23]. Ultrasound sessions in small groups of students (4 to 8 students) were in general preferred by the teachers and the students as they allow guidance in the proper probe placement, imaging acquisition and accurate identification [24].

**Table 5:** Descriptive data of medical student groups involved in anatomy learning sessions with ultrasound

Group size of medical students (original studies, *n* = 128)		
Average (Q1-Q3) Min – Max	139 (32.8-164.5) 6-1260	

Following the description of publication characteristics and student demographics, the type of anatomical regions taught with ultrasound were identified (Table 6). Ultrasound training on veins, the carotid artery, and major vessels were detected in the original studies and added to the vascular category. Ultrasound-guided injections were classified by authors as pertaining to both vascular and musculoskeletal (MSK) regions. The head and neck category involved carotid artery assessments and thyroid screenings. The renal region encompassed kidneys and bladder. The aorta category covered studies referencing the term “aorta” broadly, often implicating the abdominal aorta.

The obstetrics and gynecology category comprised studies focused on female pelvic organs and breast biopsy, while male reproductive anatomy was represented under a separate prostate and testicular category. The lung category incorporated training on pulmonary anatomy, pleural ultrasound, and pneumothorax identification. Cardiac-focused areas encompassed ultrasound of the parasternal long axis, inferior vena cava, and apical four-chamber view. The upper abdomen category included stomach, liver, gallbladder, and pancreas. Studies covering tendons, joints, ligaments, and muscles were grouped under MSK.

Vascular and cardiac anatomy were the most frequently targeted regions for ultrasound instructions(Table 6). The upper abdomen was addressed in nearly 40% of the studies, while MSK, head/neck, renal, and aorta regions were considered in approximately 30% of the studies. The least represented regions were the lungs, ocular, and prostate/testicular anatomy (Table 6).

**Table 6:** Specific area of anatomy instructed during the ultrasound sessions

Anatomical area	*n* (%) *
Vascular Cardiac Upper abdomen MSK Head/neck Renal Aorta Obstetrics/Gynecology Lung Ocular Prostate/testicular	59 (46.1) 59 (46.1) 50 (39.1) 43 (33.6) 41 (32.0) 41 (32.0) 40 (31.3) 28 (21.9) 17 (13.3) 12 (9.4) 7 (5.5)

Abbreviation: MSK, musculoskeletal

*The total (n) and total percentage (%) are above *n* = 128 and 100% as most studies include more than one anatomical area to identify

### Study and learning outcomes

Learning outcomes in the original studies were identified based on the interpretations of results and conclusions [25]. A positive learning outcome was defined in our study as a significant improvement in skills and knowledge related to ultrasound use, high student satisfaction and confidence to perform ultrasound examinations and learn anatomy, increased motivation to use ultrasound for future practice and potential long-term retention. Mixed outcomes represented progress in the learning of students for some points and an essential need to improve other points with further studies. Neutral outcomes were characterized as no shown effect or difference between groups and/or no main conclusion based on the learning outcomes of medical students. A negative outcome was defined as a decrease in skills and knowledge, increased confusion or a lack of interest in ultrasound to learn anatomy. Close to 80% of the studies reported a positive outcome when teaching anatomy with clinical ultrasound to medical students (Table 7). A negative outcome was described in two studies [26, 27], in which the ultrasound scan was the least preferred method to learn anatomy compared to lectures, textbooks, dissections, lab videos, 3-dimentional radiology [26] or other radiological imaging techniques [27].

**Table 7:** Study outcomes in anatomy learning with ultrasound (original studies, *n* = 128)

Outcome	*n* (%)
Positive Mixed Neutral Negative	100 (78.1) 16 (12.5) 10 (7.8) 2 (1.6)

Abbreviation: Nd, non-determined

Percentages may not total 100 due to rounding

According to level 1 of the Kirkpatrick´s model, the reaction to the training experience was globally positive with increased confidence and comfort levels in identifying anatomical landmarks with ultrasound [28–30]. Interpretations on the satisfaction, perception and confidence of students using ultrasound during their medical education have been broadly studied, and additional information can be found in other systematic reviews [10, 12, 25].

The reaction to the learning experience or learning outcome as level 2 of the Kirkpatrick´s model in the original studies showed mixed results. Knowledge and skills of students were generally evaluated within the same group of students at post-test or in the interval between pre- and post-test as seen in the results of this review and as explained in other systematic reviews [12, 18, 25]. Within the selected original publications in this review with a second group or a control group of students, one study reported a non-significant difference in written anatomy final examination and anatomy practical scores between the experimental group of first- and second-year medical students participating in a practical ultrasound workshop on neurologic disorders and the control group not participating in the workshop [31]. Another study with first-year medical students compared the degree of deviance in palpation of shoulder anatomical areas between a student group receiving ultrasonography instructions, materials, equipment, anatomical atlas and a control student group given the anatomical atlas only [32]. There were no significant differences in the degree of deviance between the student groups for three out of four anatomical landmarks to identify [32]. However, one study showed an improvement in the identification of cardiovascular structures for first-year medical students with ultrasound images between pre- and post-training and between the first-year medical student group with ultrasound training (intervention) and second-year medical student group without training as control [33]. In the same study, the ability to recognize anatomical structures was greater in cadaveric images compared to ultrasound images. The skills acquired by the students during the study decreased after 6 months, indicating impaired long-term retention of skills and a need for regular training [33].

The behavior defined by the authors of this review as the transfer of skills and knowledge as well as long-term retention (level 3) was demonstrated in a few original publications. Students from one study [34] agreed to the proposition that the workshop on sonographic anatomy and guided-injection with ultrasound will change how they handle the pain management of patients in their future medical practice [34]. In a similar way, another study indicated that students significantly agreed to the statement: “ultrasound will play a significant role in their future medical practice after participating in this workshop” on neurological disorders [35]. Long-term retention was rarely evaluated in the selected studies (10/128), and the results were mixed. Knowledge retention in knee anatomy with ultrasound was partially lost 9 weeks after the training in first-year medical students as students could not identify and differentiate knee pathologies in ultrasound images after 9 weeks as well as they did between pre- and post-tests [36]. The studies assessing skills or hands-on retention in identifying anatomy landmarks with ultrasound demonstrated greater long-term retention. The ability to identify fetal heart, head, placental location and vertical pocket of amniotic fluid was improved with students who participated in an obstetric workshop and were tested in their skills after 3 months compared to students who did not participate in the workshop, indicating a good retention of obstetrics hands-on ultrasound skills [37]. An improved skills retention was observed in a study conducting a flipped-classroom with a workshop for POCUS learning in cardiac anatomy [38]. Three weeks after the workshop, 26 out of 32 students could generate images of all 4 cardiac views (100% score) and label structural areas in an appropriate way during an OSCE follow-up [38]. A different study evaluated the student skills acquisition after multiple dyspnea teaching sessions and compared the student skills retention with the same instructors after 8 months [39]. There was no significant difference in the skills of students between the end of the teaching session and the retention test 8 months later, indicating good retention of skills [39]. Altogether, these results could indicate that practical knowledge of anatomy with ultrasound is better retained than theoretical knowledge. The repetition of training sessions is necessary to retain skills and knowledge [40].

Among the original studies selected for the literature review, some have implemented a standardization of educational anatomy teaching with ultrasound within an institution after years of experience [23, 41]. In both studies, courses on human anatomy with clinical ultrasound were organized for first-year medical students with anatomy blocks including practical ultrasound sessions. There is no standard method to teach anatomy with ultrasound or implement anatomy ultrasound in the medical curriculum at a national level [42]. Organizations such as the Society of Ultrasound in Medical Education and the EFSUMB exist to monitor and update POCUS knowledge as well as provide recommendations on ultrasound training in medical education [8, 9, 23].

The costs (level 4) related to the application of ultrasound in anatomy courses for medical curriculum vary to a large extent depending on the objective and design of the course. Ultrasound machines, high technology portable ultrasound devices connected to tablets and annual subscription cloud storage were estimated to several thousands of dollars [43]. Costs could be reduced with specific activities such as the small-scale production of gelatin models. For example, gelatin models can be developed to simulate breast biopsy with ultrasound and the current published recipes cost less than 5 dollars (USD) per model [44]. A study calculated the cost of implementing an anatomy and ultrasound surgical training program with embalmed cadavers and surgery supplies [45]. The total cost amounted to 716 dollars (USD) per student per year for the specific study [45].

Despite potentially high initial costs, the application of clinical ultrasound in anatomy teaching offers multiple opportunities such a direct application on real or standardized patients [22, 23, 30, 37, 39, 46], gamification and virtual/mixed reality options [43, 47, 48] and a diversity in teaching types such as lectures with PowerPoint, manual reading, online content, 3-dimensional models, near-peer teaching, team-based learning and flipped-classroom [38, 43, 48, 49]. With portable ultrasound devices, anatomy teaching can be applied in low-income countries, difficult to reach or rural areas and improve the detection rate of pathologies in remote places [46].

## Discussion

This systematic review encompassed a broad range of topics across 128 original studies investigating the application of clinical ultrasound in the medical curriculum to teach anatomy. The study shows singularity in the important selection of original publications to review and in the choice of specified and acknowledged assessment methods to optimize objectivity and reduce risks of bias when reporting information.

The overall quality of the original articles included in the review was low with an average MERSQI of 10.3 out of 18 (from 5 to 15.5). The lowest quality score was obtained for the validity of evaluation instruments´ score domain. Higher validity within the studies could be acquired with the inclusion of additional statistical analyses such as Cronbach´s alpha test to verify the validity and reliability of the tests and questionnaires conducted on medical students. Additionally, more information on the characteristics of medical students (age, gender, stage of education) and other variables depending on the study design (anatomical areas, level of ultrasound experience, ultrasound teaching methods) could be collected to better understand the factors related to the learning outcomes of medical students. Nevertheless, statistical analyses were conducted appropriately in most of the studies to compare knowledge, skills and perceptions of medical students. Objectives/subjective evaluation methods were both included in the studies, providing an important differentiation between the perceived understanding of medical students and their actual academic/practical knowledge and skills.

Several challenges related to the use of clinical ultrasound in anatomy teaching were identified in the selected studies. The lack of time to add ultrasound sessions to the medical curriculum, a lack of available and certified ultrasound instructors, a lack of equipment and resources were often reported [28]. Many studies did not include a control group in their design, the effectiveness of clinical ultrasound application in anatomy learning is therefore difficult to generalize. Studies were primarily conducted on volunteering and motivated students, which could lead to self-selection bias and might not represent a standard group of medical students. The long-term retention of knowledge and skills was not frequently evaluated in the studies and showed a low retention [33]. Practical ultrasound training sessions were canceled during the pandemic situation [23, 50]. The transfer to an online teaching method was an opportunity to develop additional sonography simulations [50–52]. Despite the large majority of students considering ultrasound technique interesting and relevant to their future practice, two studies in this review indicated ultrasound method as the least helpful imaging technique to learn anatomy compared to CT and MRI due to its complexity [26, 27]. Another important barrier to consider is the regulation of patient health data protection and security as a result of an increase in technological device application for teaching purposes in medicine [53].

### Limitations

This systematic review presents multiple limitations with the possibility of missing relevant publications. To minimize this effect, three different databases were searched. Because of the high heterogeneity of studies relevant to the subject retrieved after the systematic search, the authors decided to describe a global approach of the anatomy teaching environment with clinical ultrasound and not to specialize in a single method. The variability of the publications in design, teaching methods and outcome studied constitutes a challenge to generalize and standardize teaching. This diversity could be attributed to the versatility of ultrasound applications. The variety of designs in anatomy teaching with clinical ultrasound is an opportunity to reach a large number of medical students in other clinical fields, to adapt to different audiences, budgets and institutions.

## Conclusion

The findings of this review underscore the diverse teaching formats and educational opportunities associated with ultrasound-based anatomy instruction. The included studies were highly heterogeneous in design and content, making it difficult to draw generalized conclusions. To better evaluate the effectiveness of ultrasound in anatomy education, more standardized and objective assessment tools are needed. Furthermore, additional research is warranted to investigate the long-term retention of anatomical knowledge and skills acquired through ultrasound training. Given its clinical relevance, the integration of ultrasound into the medical curriculum holds significant potential for enhancing practical competencies of medical students and ultimately, improving patient care.

## Supplementary Information

Below is the link to the electronic supplementary material.

Supplementary Material 1

## Data Availability

The dataset generated for this study is available in the manuscript and supplementary information.

## Abbreviations

CT: Computer Tomography
EFSUMB: European Federation of Societies for Ultrasound in Medicine and Biology
MERSQI: Medical Education Research Study Quality Instrument
MRI: Magnetic resonance imaging
MSK: Musculoskeletal
Nd: Non-determined
OSCE: Objective Structured Clinical Examination
PRISMA: Preferred Reporting Items for Systematic reviews and Meta-Analyses
