# Supplementary material for: Bringing anatomy to life: the role of clinical ultrasound in undergraduate medical education – a systematic review
Source: Ultrasound J. 2025 Aug 7;17:39. doi: 10.1186/s13089-025-00443-3 (PMC12331568; doi:10.1186/s13089-025-00443-3)
Supplement: Supplementary file 1 — Supplementary Material 1 [file 13089_2025_443_MOESM1_ESM.pdf]

Appendix S1. List of selected articles for the systematic review on clinical ultrasound application to teach anatomy in medical education (N=194)

Authors	Title	Year	Medical year	Sample size	Course duration	Country
Adrian et al.	Teaching module on ultrasound-guided venous access using a homemade gel model for fourth-year medical students	2022	4	150	30 minutes	USA
Ahn et al.	Using ultrasound to enhance medical students' femoral vascular physical examination skills	2015	2	150	nd	USA
Alerhand et al.	Integrating basic and clinical sciences using Point-of-care renal ultrasound for preclerkship education	2020	1	31	2 hours	USA
Allsop et al.	Implementing ultrasound sessions to highlight living anatomy for large medical student cohorts	2021	2	250	over a year	UK
Al-Redouan et al.	Visible Human Project based applications can prompt integrating cross-sectional anatomy into the medical school curriculum when combined with radiological modalities: a three-year cross-sectional observational study	2025	1	50	20 hours	Czech Republic
Altersberger et al.	Student perceptions of instructional ultrasound videos as preparation for a practical assessment	2019	4	445	nd	Austria
Aquino et al.	Comparing peer-taught student tutors to faculty-taught student tutors in educating medical students on musculoskeletal ultrasound	2024	1	60	1 hour	USA
Atalla et al.	Investigating the skill development of medical students in focused assessment	2023	1,2	71	3 hours	Canada, UK

	with sonography for trauma (FAST) ultrasound: a comparative analysis across different stages of medical training					
Baatar et al.	Development of a competency-based training in obstetrics and gynecology ultrasound for undergraduate and graduate medical education	2014	nd	nd	nd	USA
Bagley et al.	A comparison of sonography and radiography student scores in a cadaver anatomy class before and after the implementation of synchronous distance education	2015	nd	462	nd	USA
Baltarowich et al.	National ultrasound curriculum for medical students	2014	nd	nd	4 years	USA
Barrington et al.	Determining the learning curve for acquiring core sonographic skills for ultrasound-guided axillary brachial plexus block	2016	3,4	10	5,5 hours	Australia
Barry et al.	The clinical anatomy and imaging laboratory: vertical integration in the preclerkship curriculum	2019	1,2	300	nd	USA
Barth et al.	Student ultrasound education, current views and controversies; who should be teaching?	2024	nd	nd	nd	Germany/Switzerland
Bauman et al.	An interprofessional senior medical student preparation course: improvement in knowledge and self-confidence before entering surgical training	2021	nd	22	105 hours	USA
Beck et al.	Cardiac ultrasound training for medical students utilizing drawing and ward-based instruction	2025	5	121	2 weeks	New Zealand

Bell et al.	Using ultrasound to teach medical students cardiac physiology	2015	1	20	45 minutes	USA
Bell et al.	Active learning of the floor of mouth anatomy with ultrasound	2019	1	33	7 hours	USA
Benninger.	Google Glass, ultrasound and palpation: the anatomy teacher of the future?	2015	1	106	over a year	USA
Benninger et al.	Classic versus millennial medical lab anatomy	2014	nd	nd	12 weeks	USA
Ben-Sasson et al.	Peer-teaching cardiac ultrasound among medical students: a real option	2019	1	66	8 hours	Israel
Bilella et al.	Body painting, ultrasound, clinical examination, and peer-teaching: a student-centered approach to enhance musculoskeletal anatomy learning	2024	2	60	8 days	Switzerland
Birrane et al.	A scoping review of ultrasound teaching in undergraduate medical education	2018	nd	nd	nd	Ireland/Canada
Blackstock & Carmody.	Transforming learning anatomy: basics of ultrasound lecture and abdominal ultrasound anatomy hands-on session	2016	1	134	2 hours	USA
Boivin et al.	Evaluation of a required vertical point-of-care ultrasound curriculum for undergraduate medical students	2022	1 to 4	439	37 hours	USA
Boulger et al.	ITSUS: Integrated, Tiered, Self-directed Ultrasound Scanning for learning anatomy	2021	nd	nd	nd	USA
Bowman et al.	The use of real time ultrasound scanning as a teaching method of anatomy in an undergraduate sonography and medical imaging degree in an Australian university	2016	1	34	nd	Australia
Brackney et al.	The utility of cardiac ultrasound in preclinical medical school curriculum	2016	1	54	6 hours	USA

Bretagne et al.	Randomized controlled study of a training program for knee and shoulder arthrocentesis on procedural simulators with assessment on cadavers	2022	4,5	29	nd	France
Brown et al.	Musculoskeletal ultrasound training encourages self-directed learning and increases confidence for clinical and anatomical appreciation of first-year medical students	2022	1	280	23 weeks	USA
Buhumaid et al.	From classroom to bedside: the role of point-of-care ultrasound in undergraduate medical education	2024	nd	nd	nd	UAE
Carter et al.	Integration of 3D/4D ultrasound in teaching medical anatomy	2016	1	108	100 minutes	USA
Carter et al.	Thyroid gland visualization with 3D/4D ultrasound: integrated hands-on imaging in anatomical dissection laboratory	2017	1	108	20 minutes	USA
Cecilio-Fernandes et al.	The effects of expert and augmented feedback on learning a complex medical skill	2020	2,3,4,6	36	2-3 hours	Netherlands
Chan et al.	The role of ultrasound and preparation in first-year medical students' learning lumbar spine anatomy and diagnostic skills	2017	1	318	2 hours	USA
Chang Chan et al.	Radiological anatomy as an alternative approach in anatomy teaching. Perception and performance of medical students	2022	3,6	154	30 hours	Nicaragua
Chen et al.	Does ultrasound education improve anatomy learning? Effects of the Parallel Ultrasound Hands-on (PUSH) undergraduate medicine course	2022	3	140	8,5 hours	Taiwan

Cohen et al.	Obstetric-focused POCUS training for medical students	2023	1,2	8	1 hour	USA
Cook et al.	Simulation-based clinical learning for the third year medical student: effectiveness of transabdominal and transvaginal ultrasound for elucidation of OB/GYN scenarios	2020	3	68	1 hour	USA
Corrêa et al.	Early incorporation of ultrasound into the medical curriculum through its association with human anatomy	2022	5,6	33	6 hours	Brazil
Cowan et al.	Ultrasound in medical education: can students teach themselves?	2021	nd	21	100 minutes	USA
Danila et al.	Team-based learning & point of care ultrasound (POCUS) to augment a preclinical cardiovascular physiology course	2024	1	54	115 minutes	USA
Darici et al.	"Fun slipping into the doctor's role"- The relationship between sonoanatomy teaching and professional identity formation before and during the Covid-19 pandemic	2022	2	1326	nd	Germany
Darici et al.	Medical imaging training with eye movement modeling examples: a randomized controlled study	2023	2	106	20 minutes	Germany
de Lange et al.	Wireless ultrasound devices in anatomy education: insights from medical undergraduates	2024	3	283	nd	South Africa
de Vries et al.	Effect of ultrasonography on student learning of shoulder anatomy and landmarks	2018	1	64	5 hours	USA

Denny et al.	Ultrasound curriculum taught by first-year medical students: a four-year experience in Tanzania	2018	1	309	4 weeks	USA
DesJardin et al.	A near-peer point-of-care ultrasound elective for medical students: impact on anatomy knowledge, perceptions about ultrasound, and self-reported skill level	2017	1	38	5 months	USA
Dickerson et al.	The role for peer-assisted ultrasound teaching in medical school	2017	5	103	nd	UK
Ding et al.	The effect of simulation of sectional human anatomy using ultrasound on students´learning outcomes and satisfaction in echocardiography education: a pilot randomized controlled trial	2024	1	18	1 hour	China/USA
Dissaux et al.	Assessment of using ultrasonography to teach abdominal and cervical anatomy in French medical curricula	2023	5	112	nd	France
Donald et al.	Putting the cart before the horse? Developing a blended anatomy curriculum supplemented by cadaveric anatomy	2023	nd	nd	nd	UK
Draper et al.	Ultrasound education in an elective course in the undergraduate medical curriculum improves students´subjective learning and clinical rotation and residency preparedness	2023	3,4	201	nd	USA
Dreher et al.	Ultrasound exposure during gross anatomy	2014	1	269	5,5 hours	USA
Ertl-Wagner et al.	White paper: radiological curriculum for undergraduate medical education in Germany	2016	nd	nd	nd	Germany

Fakoya et al.	Ultrasound and stethoscope as tools in medical education and practice: considerations for the archives	2016	nd	nd	nd	Grenada
Feilchenfeld et al.	Ultrasound in undergraduate medical education: a systematic and critical review	2017	nd	nd	nd	Canada
Fenech et al.	Collaborative use of a 3D anatomy platform to motivate and enhance anatomy learning in first-year online medical sonography students	2024	1	61	nd	Australia
Galusko et al.	Hand-held ultrasonography: an opportunity for "hands-on" teaching of medicine	2016	1,2	40	7 hours	UK
Garrido et al.	Development of a radiology curriculum for undergraduate medical education: experience of a School of Medicine of Chile	2018	nd	nd	6 years	Chile
Ghosh.	Cadaveric dissection as an educational tool for anatomical sciences in the 21st century	2017	nd	nd	nd	India
Goldstein et al.	Efficacy of a point-of-care transthoracic echocardiography workshop for medical students	2021	2,3	8	5 weeks	USA
Gradl-Dietsch et al.	Multidimensional approach to teaching anatomy - Do gender and learning style matter?	2016	2	360	165 minutes	Germany
Graziani et al.	Technologies for studying and teaching human anatomy: implications in academic education	2024	nd	nd	nd	Brazil
Grignon et al.	Teaching medical anatomy: what is the role of imaging today?	2016	nd	nd	nd	France

Haidar et al.	Association of a longitudinal, preclinical ultrasound curriculum with medical student performance	2022	1	178	15 hours	USA
Haji-Hassan et al.	Effectiveness of ultrasound cardiovascular images in teaching anatomy: a pilot study of an eight-hour training exposure	2022	1,2	140	8 hours	Romania
Haji-Hassan et al.	Efficacy of handheld ultrasound in medical education: a comprehensive systematic review and narrative analysis	2023	nd	nd	nd	Romania
Hamza et al.	Introduction of a student tutor-based basic obstetrical ultrasound screening in undergraduate medical education	2019	nd	111	1 day	Germany
Hamza, et al.	Trial integration of combined ultrasound and laparoscopy tuition in an undergraduate anatomy class with volunteer participation - A pilot study	2019	nd	25	3 hours	Germany
Hartup et al.	Transvaginal ultrasound simulation: educational benefits on obstetrics and gynaecology clerkship	2025	3	111	10 hours	USA
Hendi.	Effectiveness of a short course on undergraduate medical students' acquisition of basic ultrasound skills: findings from a Saudi university	2022	4	118	170 minutes	Saudi Arabia
Hennekes et al.	The PEGASUS Games: Physical Exam, Gross Anatomy, phySiology and UltraSound games for preclinical medical education	2021	1	20	2 hours	USA
Hey et al.	Simulation-based breast biopsy training using a low-cost gelatin-based breast model in Rwanda	2023	nd	28	nd	USA/Rwanda

Ho et al.	Introducing final-year medical students to pocket-sized ultrasound imaging: teaching transthoracic echocardiography on a 2-week anesthesia rotation	2015	5	133	3 hours	China
Houser & Kondrashov.	Gross anatomy education today: the integration of traditional and innovative methodologies	2018	1	172	nd	USA
Hoyer et al.	Ultrasound-guided procedures in medical education: a fresh look at cadavers	2016	3	55	2 days	USA
Hu et al.	Impact of virtual reality anatomy training on ultrasound competency development: a randomized controlled trial	2020	3	101	6 hours	Taiwan
Huettner et al.	Medical student instruction in peripheral nerve blockade utilizing fresh cadaver limbs in a simulation center	2023	1	8	6 hours	USA
Ireson et al.	First year medical students, personal handheld ultrasound devices, and introduction of insonation in medical education	2019	1	53	nd	USA
Jamniczky et al.	Cognitive load imposed by ultrasound-facilitated teaching does not adversely affect gross anatomy learning outcomes	2015	1	137	2 hours	Canada
Johnson et al.	Using two-dimensional ultrasound imaging to examine venous pressure	2020	nd	nd	nd	UK
Johnson et al.	Ultrasound technology as a tool to teach basic concepts of physiology and anatomy in undergraduate and graduate courses: a systematic review	2025	nd	nd	nd	UK
Jurjus et al.	Can anatomists teach living anatomy using ultrasound as a teaching tool?	2014	1	178	110 hours	USA

Kafer et al.	Hunger Games: interactive ultrasound imaging for learning gastrointestinal physiology	2017	1	100	nd	USA
Kailin et al.	Online learning and echocardiography boot camp: innovative learning platforms promoting blended learning and competency in pediatric echocardiography	2021	nd	6	3 days	USA
Kameda et al.	Ultrasonography in undergraduate medical education: a comprehensive review and the education program implemented at Jichi Medical University	2022	nd	nd	nd	Japan
Karim et al.	Evaluating the impact of structured POCUS training during internal medicine clerkship and residency training	2024	3,4	10	2 weeks	USA
Keenan & Powell.	Interdimensional travel: visualisation of 3D-2D transitions in anatomy learning	2020	nd	nd	nd	UK
Kefala-Karli et al.	Introduction of ultrasound-based living anatomy into the medical curriculum: a survey on medical students´perceptions	2021	1,2	149	80 minutes	Cyprus
Kenny et al.	The use of ultrasound in undergraduate medical anatomy education: a systematic review with narrative synthesis	2022	nd	nd	nd	UK/USA
Khoury et al.	Preclerkship point-of-care ultrasound: image acquisition and clinical transferability	2020	1,2	32	5 hours	Canada
Kim et al.	Educational value of pocket-sized ultrasound devices to improve understanding of ultrasound examination principles and sonographic anatomy for medical student	2017	1	40	nd	Korea

Kim et al.	Ultrasonographic images and correspondence with real color sectioned images of the upper limb	2024	nd	nd	nd	Korea
Knapp et al.	The evolution of ultrasound in medicine: a case report of point-of-care ultrasound in the self-diagnosis of acute appendicitis	2020	1	1	nd	USA
Knudsen et al.	Hands-on or no hands-on training in ultrasound imaging: a randomized trial to evaluate learning outcomes and speed of recall of topographic anatomy	2018	1	43	5 hours	Germany
Kochhar et al.	Is cadaveric dissection essential in medical education? A qualitative survey comparing pre-and post-COVID-19 anatomy courses	2023	1	149	8 weeks	USA
Kondrashov et al.	Impact of the clinical ultrasound elective course on retention of anatomical knowledge by second-year medical students in preparation for board exams	2015	2	248	nd	USA
Kondrashova et al.	Development of competency in needle-guided procedures through the use of soft-embalmed cadavers	2020	2	13	nd	USA
Kondrashova & Kondrashov.	Integration of ultrasonography into the undergraduate medical curriculum: seven years of experience	2018	1,2	nd	nd	USA
Kondrashova & Lockwood.	Innovative approach to teaching osteopathic manipulative medicine: the integration of ultrasonography	2015	1,2	348	2 hours	USA
Koratala.	Nephrologist-led simulation-based focused cardiac ultrasound workshop for medical students: insights and implications	2023	4	25	4 hours	USA

Lewiss et al.	Point-of-care ultrasound education: the increasing role of simulation and multimedia resources	2014	nd	nd	nd	USA
Li et al.	A hands-on organ-slicing activity to teach the cross-sectional anatomy	2020	3	182	90 minutes	China
Liao et al.	Augmented reality visualization for ultrasound-guided interventions: a pilot randomized crossover trial to assess trainee performance and cognitive load	2024	6	22	nd	Taiwan
Lorke, et al.	Creation of 21st century anatomy facilities: designing facilities for integrated preclinical education in the Middle East	2023	nd	79	nd	UAE
Luetmer et al.	Simulating the multi-disciplinary care team approach: enhancing student understanding of anatomy through an ultrasound-anchored interprofessional session	2018	1	53	120 hours	USA
Lufler et al.	Bringing anatomy to life: evaluating a novel ultrasound curriculum in the anatomy laboratory	2022	1	211	20 weeks	USA
Makris.	Simulation in radiology education	2022	nd	nd	nd	USA
Maloney et al.	Use of gelatin puzzle phantoms to teach medical students isolated ultrasound transducer movements and fundamental concepts	2020	nd	162	55 minutes	USA
Mancine et al.	Discovering pathologies in the anatomy lab: the case of brachial plexopathy mimicking neurological thoracic outlet syndrome	2020	nd	1	nd	USA
Margenfeld et al.	The feasibility of ultrasound-guided latex labeling of the anterolateral	2024	nd	128	nd	Switzerland/Germany

	ligament in anatomical dissection - A cadaveric study					
Margenfeld et al.	Review of ultrasound-guided labeling: exploring its potential in teaching cadaveric ligaments during anatomical dissection courses	2024	nd	nd	nd	Switzerland
Margenfeld et al.	The advantages of utilizing different ultrasound imaging techniques on joints of human cadavers in the teaching of anatomy - A scoping review	2024	nd	nd	nd	Switzerland
Martin et al.	The rising tide of point-of-care ultrasound (POCUS) in medical education: an essential skillset for undergraduate and graduate medical education	2023	nd	nd	nd	USA
Mason et al.	Two affordable, high-fidelity central venous models for ultrasound-guided interventional training	2024	1 to 3	20	2 hours	USA
McCormick et al.	Ultrasound skills teaching in UK medical education: a systematic review	2023	nd	nd	nd	UK
McCrary et al.	Development of a fresh cadaver model for instruction of ultrasound-guided breast biopsy during the surgery clerkship: pre-test and post-test results among third-year medical students	2016	3	41	nd	USA
McCrary et al.	A fresh cadaver model for the instruction of ultrasound-guided fine-needle aspiration of thyroid nodules	2017	1,2	17	nd	USA
McKinley et al.	Utilizing 3-dimensional cardiac models with point-of-care ultrasound video tutorials to improve medical student	2023	1,2	39	20 minutes	USA

	education: a double-blinded randomized control study					
Meuwly et al.	Use of an online ultrasound simulator to teach basic psychomotor skills to medical students during the initial COVID-19 lockdown: quality control study	2021	1	193	3,5 months	Switzerland
Michaelis et al.	Looking through the crystal ball feasibility of tele-echocardiography using smart glasses in neonates: a pilot study	2025	nd	21	nd	Germany
Miller et al.	Learner improvement from a simulation-enhanced ultrasonography curriculum for first-year medical students	2017	1	390	195 minutes	USA
Minardi et al.	Longitudinal ultrasound curriculum incorporation at West Virginia University School of Medicine: a description and graduating students' perceptions	2019	4	82	nd	USA
Miner et al.	Feasibility study of first-year medical students identifying cardiac anatomy using ultrasound in rural Panama	2015	1	7	12 hours	USA/Panama
Moga et al.	Learning curves in abdominal ultrasound in medical students	2024	4, 5	93	28 hours	Romania
Moscova et al.	Integration of medical imaging including ultrasound into a new clinical anatomy curriculum	2015	1,2	875	nd	Australia
Muench & Schellpfeffer.	Qualitative evaluation of incorporating ultrasound education into an undergraduate medical education clinical human anatomy course	2024	1	63	3,5 hours	USA

Nausheen et al.	Confidence level and ability of medical students to identify abdominal structures after integrated ultrasound sessions	2020	1	25	nd	USA
Nausheen et al.	Impact of multimodality integrated anatomy teaching approach towards teaching effectiveness, student engagement, and social interaction	2021	1	88	nd	USA/Egypt
Nelson et al.	Including insonation in undergraduate medical school curriculum	2019	nd	nd	nd	USA
Nithipalan et al.	Evaluation of mixed reality technologies for remote feedback and guidance during transrectal ultrasound biopsy simulation: a prospective, randomized, crossover study	2024	nd	19	3,5 hours	USA
Noël et al.	3D printed heart models illustrating myocardial perfusion territories to augment echocardiography and electrocardiography interpretation	2021	1	183	1 hour	Canada
Olivares-Perez et al.	Virtual anatomy and point-of-care ultrasonography integration pilot for medical students	2022	1	161	2,5 hours	USA
O'Reilly et al.	Fabrication and assessment of 3D printed anatomical models of the lower limb for anatomical teaching and femoral vessel access training in medicine	2016	nd	nd	nd	Ireland
Paganini & Rubini.	Ultrasound-based lectures on cardiovascular physiology and reflexes for medical students	2016	2	111	45 minutes	Italy
Palma.	Successful strategies for integrating bedside ultrasound into undergraduate medical education	2015	nd	nd	nd	USA

Parikh et al.	Novel use of ultrasound to teach reproductive system physical examination skills and pelvic anatomy	2018	2	96	8 hours	USA
Patel et al.	Integrating ultrasound into modern medical curricula	2017	nd	nd	nd	New Zealand/USA
Patra et al.	Blending conventional learning approach with contemporary visualization technologies: prerequisite toward evidence-based surface anatomy	2024	nd	nd	nd	India
Patten.	Using ultrasound to teach anatomy in the undergraduate medical curriculum: an evaluation of the experiences of tutors and medical students	2015	1,2	436	2 hours	UK
Pazeli et al.	Point-of-care ultrasound evaluation and puncture simulation of the internal jugular vein by medical students	2018	4	6	4 hours	Brazil
Petty et al.	Performance assessment in medical school curricula: an innovative method of evaluating sonographic skills using ultrasound practical examinations	2016	1	537	max 1 hour	USA
Potter et al.	Generative retrieval does not improve long-term retention of regional anesthesia ultrasound anatomy in unengaged learners	2019	4	45	47 minutes	USA
Prager et al.	Point of care ultrasound in undergraduate medical education: a survey of University of British Columbia medical student attitudes.	2016	nd	59	4 hours	Canada
Rajendram et al.	Investigating medical students´ perceptions of point-of-care ultrasound integration into preclinical education	2024	6	229	nd	Saudi Arabia

Rathbun et al.	Incorporating ultrasound training into undergraduate medical education in a faculty-limited setting	2023	1,4	253	4 weeks	Greece/USA
Reed & Dennis.	Development of a cadaveric breast ultrasound module and analysis of its effectiveness among medical students	2024	1,2	104	1 hour	USA
Rempell et al.	Pilot point-of-care ultrasound curriculum at Harvard Medical School: early experience	2016	1,2	214	1-2 hours	USA
Russell et al.	Development and implementation of a point of care ultrasound curriculum at a multi-site institution	2021	1 to 4	nd	nd	USA
Russell et al.	Assessment of medical students' ability to integrate point-of-care cardiac ultrasound into a case-based simulation after a short intervention	2022	2	132	45 minutes	USA
Salewski et al.	The impact of 3D printed models on spatial orientation in echocardiography teaching	2022	5	153	4 hours	Germany
Sambi et al.	Pilot project: does formal bedside training of medical students with a FAST exam increase their knowledge and comfort level with ultrasound use in a community family medicine practice setting?	2017	3	18	3 hours	Canada
Santos et al.	Technological resources for teaching and learning about human anatomy in the medical course: systematic review of literature	2022	nd	nd	nd	Brazil
Schober et al.	Anatomy and imaging: 10 years of experience with an interdisciplinary teaching project in preclinical medical	2014	nd	618	12 hours	Germany

	education - From an elective to a curricular course					
Seijas et al.	Direct measurements of the tendon	2017	3	6	nd	Spain
Serrao et al.	Virtual dissection by ultrasound: probe handling in the first year of medical education	2017	1	653	170 hours	Italy
Sevak et al.	Solid, cystic, and tubular: novice ultrasound skills training using a versatile, affordable practice model	2018	3, 4	34	15 minutes	USA
Shah et al.	Teaching neurological disorders with ultrasound: a novel workshop for medical students	2020	1	22	2 hours	USA
Shah et al	Ultrasound's impact on preclinical medical student neurology unit grades: findings after 2 years	2021	1,2	360	1 hour	USA
Shokoohi et al.	An experiential learning model facilitates learning of bedside ultrasound by preclinical medical students	2016	1,2	90	over a year	USA
Singer et al.	Performance of an interactive upper extremity peripheral nerve training module among medical students, radiology residents, and fellows: a multi-institutional study	2020	1 to 4	20	15 minutes	USA
Situ-LaCasse et al.	Performance of ultrasound-guided peripheral nerve blocks by medical students after one-day training session	2019	3	94	1 day	USA
Smith et al.	Improved medical student perception of ultrasound using a paired anatomy teaching assistant and clinician teaching model	2018	1	365	5 hours	USA

So et al.	Ultrasound imaging in medical student education: impact on learning anatomy and physical diagnosis	2017	nd	nd	nd	USA
Steinmetz et al.	Acquisition and long-term retention of bedside ultrasound skills in first-year medical students	2016	1	195	6 hours	Canada
Stone-McLean et al.	Developing an undergraduate ultrasound curriculum: a needs assessment	2017	nd	82	nd	Canada
Teichgräber et al	Integration of ultrasonography training into undergraduate medical education: catch up with professional needs	2022	3,6	nd	nd	Germany
Torralba et al.	Teaching of clinical anatomy in rheumatology: a review of methodologies	2015	nd	nd	nd	USA/Mexico
Torres et al.	Bridging the gap between basic and clinical sciences: a description of a radiological anatomy course	2016	4	273	6 hours	Poland/USA
Trelease.	From chalkboard, slides, and paper to e-learning: how computing technologies have transformed anatomical sciences education	2016	nd	nd	nd	USA
Trembley & Radomski.	Use of ultrasound in introducing anatomical pathology to preclinical medical students, in correlation with physical exam curricula	2020	1,2	12	8 hours	USA
Truong et al.	Exploring heart dissection techniques for enhancing anatomical education: a pilot study to replicate transthoracic echocardiography	2024	2	nd	3 hours	Japan
Tullavardhana & Rookkachart.	An educational course for the achievement of confidence in basic Focused Assessment with Sonography in	2017	6	120	7 hours	Thailand

	Trauma (FAST): evaluation of a small group workshop in Thai medical student					
Umar et al.	Real time sonography as an anatomy teaching aid in undergraduate radiography institutions in Northern Nigeria	2019	3 to 5	92	nd	Nigeria
Valenciaga et al.	Efficacy of an integrated hands-on thyroid ultrasound session for medical student education	2021	2	209	1 hour	USA
Vandenbossche et al.	Ultrasound versus videos: a comparative study on the effectiveness of musculoskeletal anatomy education and student cognition	2023	2	181	75 hours	Belgium
Vandenbossche et al.	Anatomical knowledge enhancement through echocardiography and videos, with a spotlight on cognitive load, self-efficacy, and motivation	2024	2	206	135 minutes	Belgium
Vaudrey et al.	The impact of designing near-peer teacher training according to Merrill´s First Principles of Instruction	2024	2	10	nd	USA
Vollman et al.	Educational benefits of fusing magnetic resonance imaging with sonograms	2014	2,3	31	nd	USA
Wakefield et al.	The development of a pragmatic, clinically driven ultrasound curriculum in a UK medical school	2018	1 to 5	nd	nd	UK
Walrod et al.	Does ultrasound-enhanced instruction of musculoskeletal anatomy improve physical examination skills of first-year medical students?	2018	1	27	30 minutes	USA
Walrod et al.	Beyond bones: assessing whether ultrasound-aided instruction and practice	2019	1	49	30 minutes	USA

	improve unassisted soft tissue palpation skills of first-year medical students					
Wang et al.	Why do pre-clinical medical students learn ultrasound? Exploring learning motivation through ERG theory	2021	3	140	nd	Taiwan
Weaver et al.	Effects of a focused training on first-year osteopathic medical students' ability to incorporate point-of-care ultrasound in assessment of the anterior knee	2023	1	101	1,5 hours	USA
Webb et al.	Teaching point of care ultrasound skills in medical school: keeping radiology in the driver's seat	2014	1	154	1,5 hours	USA
Weimer et al.	FoCUS cardiac ultrasound training for undergraduates based on current national guidelines: a prospective, controlled, single-center study on transferability	2023	2 to 5	217	1 day	Germany
Weimer et al.	Development and evaluation of a point-of-care ocular ultrasound curriculum for medical students - A proof-of-concept study	2023	3 to 6	33	4,5 hours	Germany
Weimer et al.	Effects of undergraduate ultrasound education on cross-sectional image understanding and visual-spatial ability - A prospective study	2024	3	141	15 hours	Germany
Weiskittel et al.	Team-based ultrasound Objective Structured Practice Examination (OSPE) in the anatomy course	2021	1	52	7 weeks	USA
Wilson et al.	Implementation of a 4-year point-of-care ultrasound curriculum in a liaison committee on Medical Education-Accredited US Medical School	2017	4	84	4 years	USA

Wilson et al.	Understanding physiology with ultrasound	2023	nd	nd	nd	USA
Wlodarkiewicz et al.	Educational value of ultrasonography used as a diagnostic tool by medical students at a student-run free clinic	2020	1,2	7	nd	USA
Zeitouni et al.	Integration of point of care ultrasound into undergraduate medical education at Texas Tech University Health Sciences Center school of medicine: a 6 year review	2024	1	1260	140 minutes	USA

Abbreviations: UAE, United Arab Emirates; UK, United Kingdom; USA, United States of America
